# Objective-function-guided automated VMAT planning reduces OAR dose, low-dose exposure, and inter-planner variability in breast radiotherapy

**DOI:** 10.1038/s41598-026-52616-2

**Published:** 2026-05-21

**Authors:** Hannes Rennau, Guido Hildebrandt

**Affiliations:** Department of Radiation Oncology, University Hospital Rostock, Rostock, 18057 Germany

**Keywords:** Cancer, Computational biology and bioinformatics, Medical research, Oncology

## Abstract

This study presents and evaluates an automated volumetric modulated arc therapy (VMAT) planning framework for breast cancer based on objective function value (OFV)–guided optimization. The primary objective is to systematically improve organ-at-risk sparing through automated and reproducible optimization of planning constraints while maintaining clinically acceptable target coverage. An OFV-guided optimization workflow was empirically developed using a separate sensitivity dataset and subsequently evaluated in 20 clinical breast cancer patients (13 left-sided, 7 right-sided). The automated Python-based framework iteratively adapts MaxEUD constraints during optimization until dose metrics converge, without manual intervention. Automatically generated plans were compared to clinically delivered VMAT plans using target coverage, dose–volume metrics, and monitor units as a surrogate for delivery efficiency. The automated approach consistently achieved significant reductions in mean organ doses and low-dose volumes (e.g., $V_{2Gy}$, $V_{5Gy}$) while preserving PTV coverage. Mean heart dose decreased from $3.09 \pm 1.06$ Gy to $2.21 \pm 0.69$ Gy for left-sided cases and from $1.88 \pm 1.06$ Gy to $1.17 \pm 0.25$ Gy for right-sided cases ($p < 0.001$). Significant dose reductions were also observed for the ipsilateral lung, contralateral lung, and contralateral breast, accompanied by a 16.9 % reduction in monitor units ($p < 0.001$) and reduced inter-patient variability. In contrast, no statistically significant difference was observed for lung $V_{20Gy}$. In conclusion, OFV-guided VMAT optimization enables reproducible and systematic improvement of organ-at-risk sparing in breast radiotherapy. By reducing organ doses, low-dose burden, monitor units, and inter-patient variability without compromising target coverage, the proposed framework could provide a robust and standardized baseline for clinical VMAT planning and a consistent foundation for future data-driven and machine-learning–based optimization approaches.

## Introduction

For several decades, the tangential 3D-conformal radiation therapy (3DCRT) technique has been the standard treatment approach for early-stage breast cancer. Several key factors have contributed to this technique remaining the standard of care, including: (i) safe dose delivery using only two angles, (ii) a wide safety margin to accommodate breathing and breast swelling, (iii) a steep dose gradient towards the ipsilateral lung, heart, and contralateral breast, and (iv) significantly fewer monitor units (MU) and a reduced low-dose burden compared to multi-field intensity-modulated radiotherapy (IMRT) or volumetric-modulated arc therapy (VMAT) techniques. However, the introduction of VMAT has significantly improved the sparing of nearby organs and treatment efficiency^1^.

Despite these advantages, fluence-modulated techniques like VMAT increase the low-dose burden to the patient’s body. This may not always justify the elevated secondary cancer risk, especially for younger women who are more vulnerable. Studies^2–4^ have argued that radiation-induced cancer risk can be reduced by using classical 3DCRT, which results in less accelerator head scatter. According to^5^, most secondary tumors occur within 5 cm of the target volume at doses below 6 Gy. Therefore, maximal protection of nearby organs must be prioritized when choosing VMAT over 3DCRT. A recent summary by^4^ even suggested the superiority of 3DCRT for early-stage cases, which motivated our investigation into whether the full potential of VMAT is currently being utilized.

Modern linear accelerators with dynamic adaptations of gantry speed and dose rate continue to enhance VMAT plan quality. VMAT has been found superior to IMRT regarding target coverage and treatment time^6–8^. While^9^ found VMAT superior to 3DCRT for heart and LAD sparing, they reported higher doses to the contralateral side. VMAT is often favored for complex plans to reduce heart dose^10^ and is superior for target coverage and ipsilateral sparing^11^. Further studies^12^ showed that VMAT yields lower heart doses than forward-planned IMRT (FIMRT). For bilateral breast cancer, hybrid approaches (h-VMAT) may be superior^13^, and VMAT remains the optimal choice for atypical anatomies like funnel chest^14^.

Many institutions rely on predefined internal optimization criteria or a specific treatment protocol^15–22^. However, while plan quality is extensively compared, technical details concerning the automation protocols themselves are often limited. In^17^, limited optimization led to VMAT heart doses nearly twice as high as for 3DCRT. This suggests that optimization is often terminated once internal, institution-specific constraints are met, potentially leaving further dose reduction untapped. Current literature indicates that while average ipsilateral lung and heart doses are often similar between 3DCRT and VMAT, contralateral doses and low-dose volumes typically remain higher with VMAT. We hypothesize that these volumes can be significantly reduced to levels approaching 3DCRT through more rigorous optimization. Motivated by these inconsistencies, we developed a method to generalize VMAT planning to systematically lower mean organ doses. While deep inspiration breath-hold (DIBH) can further reduce doses^11,23–27^, our study investigates if substantial organs at risk (OARs) reductions are achievable via OFV-guided (objective function value) planning alone.

Automated treatment planning has matured significantly, utilizing diverse strategies to reduce inter-planner variability and improve efficiency^28,29^. In modern treatment planning systems (TPS) such as RayStation and Eclipse, powerful scripting interfaces (Python) and the Eclipse Scripting API (ESAPI) allow for the implementation of complex automation frameworks. These methodologies range from rule-based heuristic scripts that mimic the decision-making process of an experienced planner to more advanced lexicographic optimization and automated multi-criteria optimization (MCO) navigation^30,31^. Scripting is also increasingly used to integrate Knowledge-Based Planning (KBP) and deep learning-based dose predictions into the clinical workflow, automatically translating predicted dose-volume histograms (DVH) into deliverable optimization objectives.

The literature consistently shows that automated and scripted treatment planning achieves target coverage comparable to manual planning while improving efficiency and reproducibility^32–36^. presented a straightforward method for automatic treatment planning using Python in RayStation, with automatically generated plans being comparable or superior to manual ones. Across multiple sites, automated plans are often more conformal and yield reduced mean doses to most OARs^32,37,38^. A key advantage is reduced inter-planner variability and planning time, enabling even less experienced planners to generate clinically acceptable plans rapidly^39,40^. However, several studies report systematic increases in monitor units and, in some cases, slightly higher low-dose exposure to selected OARs, indicating a trade-off inherent to some auto-planning strategies^39,41,42^. Comparative evaluations of different automated treatment planning systems show that most approaches meet standard DVH constraints within short effective working times, with only minor dosimetric differences between different commercially available systems^33^.

Recent advancements in automated planning have seen the integration of deep learning (DL) models into commercial treatment planning systems like RayStation. These approaches, such as the dose prediction models developed by^43^ and the clinical evaluation by^44^, have shown significant potential in streamlining workflows and mimicking expert-level dose distributions. For instance^45^, demonstrated that DL-based auto-planning for complex breast cancer cases could achieve reductions in mean heart dose (MHD) and mean ipsilateral lung dose (MLD) of up to 0.5 Gy compared to manual planning, while significantly reducing the planning time. However, these data-driven methods are inherently dependent on the quality of the underlying training datasets.

Fully automated VMAT systems have expanded to various sites, including locally advanced rectal cancer^46^ and lung cancer^47^. Recent innovations include deep-learning-assisted fluence prediction for IMRT^48^, scripted optimization frameworks^30^, and intelligent planning robots for SBRT^31^ and even WBI ^40^. However, a critical limitation remains the reliance on clinical training data representing plans optimized only until institutional treatment protocols are satisfied. Deep learning models and KBP systems are inherently limited by the quality of the plans they are trained on^49^. Pareto-optimal training datasets that systematically explore the trade-off surface enable more robust predictions^50,51^, but few clinical approaches provide a mathematical indicator of when the physical limits of dose sparing have been reached. Overall, automation is well established as a robust complement to manual planning, offering clear workflow and consistency benefits while still requiring expert oversight during model or template setup^34^. Our work extends this philosophy by using OFV-guided optimization within a scripted Python environment to systematically explore the boundary between OAR sparing and target coverage. Unlike conventional automated planning, which terminates once predefined clinical goals are fulfilled, the OFV-guided approach continues the optimization until mathematical saturation is reached, thereby systematically exploring the optimization space.

## Materials and methods

### Patient selection and technical equipment used

Twenty patients with early-stage breast cancer (13 left-sided, 7 right-sided) who had been treated with VMAT between January and Febrary 2021 at Department of Radiation Oncology (University of Rostock) were randomly selected for this retrospective study. All patients underwent CT simulation with a slice thickness of 3 mm. Three-dimensional patient models, including the planning target volume (PTV) and OARs, were originally contoured by two different physicians as part of routine clinical practice. The clinically delivered treatment plans were created by six different physicists from our department.

The average volumes (mean ± standard deviation) were as follows: PTV: $1039 \pm 495~\textrm{ccm}$ (range: $401-1946~\textrm{ccm}$), heart: $560 \pm 125~\textrm{ccm}$ ($412-950~\textrm{ccm}$), contralateral breast: $970 \pm 466~\textrm{ccm}$ ($350-1877~\textrm{ccm}$), left lung: $1514 \pm 628~\textrm{ccm}$ ($755-2294~\textrm{ccm}$), and right lung: $1852 \pm 673~\textrm{ccm}$ ($1118-3020~\textrm{ccm}$).

Treatment planning followed a standardized technical setup for both clinical reference plans and automated plans. This included (i) a prescribed dose of $50~\textrm{Gy}$ to the PTV delivered in $2~\textrm{Gy}$ fractions, (ii) two coplanar VMAT arcs with $6~\textrm{MV}$ photons (left-sided: $180^\circ$–$288^\circ$, right-sided: $180^\circ$–$72^\circ$), (iii) the definition of optimization objectives and dose–volume constraints according to the institutional clinical protocol, and (iv) application of a breast safety margin toward the body surface using the *robust* feature in RayStation. The *robust* feature ensures adequate dose coverage in the presence of breast motion and setup uncertainties (comparable to the auto-flash functionality in other treatment planning systems).

The conventionally fractionated regimen was chosen to ensure comparability with a large body of established dosimetric and radiobiological breast cancer literature and to allow model generalizability across different fractionation schemes. Using $2~\mathrm{Gy}$ fractions provides a standardized reference independent of specific hypofractionated schedules and avoids introducing fractionation-specific effects that are not the focus of this study.

Optimization of the clinical reference plans was manually fine-tuned by the planner in an iterative manner, guided by clinical dose constraints and individual experience, and stopped once the plan was considered clinically satisfactory. No objective-function–based saturation criterion was applied during clinical planning.

For inverse optimization in RayStation, consistent objective weighting was applied to ensure comparability between clinical reference plans and automated plans. In all plans, the optimization volume of the planning target volume ((PTV_opt, physical optimization structure) was assigned a fixed weight of 3000.

For the automated OFV-guided plans, all OAR objectives were assigned a uniform weight of 10. This choice ensured that the relative importance of OAR sparing was governed exclusively by the iterative adaptation of MaxEUD constraints based on the observed OFVs, rather than by manual adjustment of weighting factors.

In contrast, clinical reference plans were generated using the same PTV_opt weight of 3000, while OAR weights were adjusted manually by the individual planner according to clinical judgement and experience. Depending on the organ and patient anatomy, OAR weights in clinical plans typically ranged between 10 and 100, reflecting common clinical practice. No standardized weighting scheme was enforced for clinical planning.

The ipsilateral lung constraints were: mean lung dose $<10$ Gy, V_10Gy_ $<35$ %, and V_20Gy_ $<20$ %^52–56^. For the contralateral lung, no formal dose constraints are defined in current ESTRO, RTOG, or ASTRO guidelines; however, all recommend minimizing dose exposure as much as reasonably achievable. Contemporary breast radiotherapy studies and planning recommendations report contralateral lung mean doses typically below 1–2 Gy, even for IMRT and VMAT techniques^3,57^. Accordingly, in this study a contralateral lung mean dose constraint of $<2$ Gy was applied. The mean heart dose (Dmean) was constrained to $\le 2.5~\mathrm{Gy}$ ^58^. The mean dose to the contralateral breast was limited to $\le 2~\text {Gy}$ ^3,59,60^.

Statistical analysis was performed in Python (SciPy, version 1.2.3). Differences between clinical and auto-optimized plans were evaluated using a paired t-test (scipy.stats.ttest_rel) on matched samples. All tests were two-sided, and a *p*-value $< 0.05$ was considered statistically significant.

Treatment planning was performed using RayStation 11B (RaySearch Laboratories AB, Stockholm, Sweden), which enables direct access to and dynamic modification of optimization parameters through its Python-based scripting interface. All treatments were planned for delivery with an Elekta Versa HD™ (Elekta AB, Stockholm, Sweden) linear accelerator. All methods were carried out in accordance with relevant guidelines and regulations. The data used in this study originated from the research project PASSOS, which was supported by the German Federal Ministry of Education and Research (BMBF) under contract number 02NUK026. The original study protocol, including the use of patient data, was approved by the Ethics Committee of the University Medical Center Rostock. Written informed consent was obtained from all subjects prior to their inclusion in the PASSOS project.

### Maximum OFV value - sensitivity analysis method

To determine the OFV parameter that maximizes OAR sparing in breast cancer treatment plans while maintaining dose coverage in the PTV, we conducted a multi-step sensitivity analysis for ten breast cancer patient plans that were exclusively used for sensitivity analysis and were not included in the 20-patient cohort used for automatic replanning and evaluation. After the first optimization (uniform PTV dose = 50 Gy) without any explicit settings for the OAR, PTV coverage was satisfactory but adjacent organs were insufficiently spared. In the second optimization step, we reduced the ipsilateral lung mean dose to 50 % of its initial value and imposed constant maximum EUD limits for all other OARs (contralateral lung: 1 Gy, contralateral breast: 4 Gy, heart: 2.5 Gy) to preserve the tangential dose distribution while continuously improving lung sparing. In the third iteration, the ipsilateral lung dose was halved again, and in all subsequent runs it was further reduced to 80 % of the previous dose. After each run we recorded PTV coverage (PTV95 %Vol), PTV-OFV, external maximum dose, external volume > 105 % of prescription, OAR-OFV and mean OAR dose (1). To find the optimal OFV tuning point, we smoothed each metric with a three-point moving average and calculated local change rates, defined as numerical finite differences of the evaluated dose metrics between consecutive optimization steps and applied clinical “criticality” masks (PTV95 %Vol $< 47.5~\textrm{Gy}$; $|\Delta \text {mean lung dose}|$ below its median slope; Ext-max $> 52.5~\textrm{Gy}$; $\Delta (\text {Ext}> 105\,\%)$ above its median).

Summing the absolute derivatives of metrics that met these criteria yielded an aggregate sensitivity score S(j). The maximum S(j) yielded an average OFV of 2.25 over all ten patients that was chosen as the data-driven tuning point that maximizes organ sparing without compromising PTV coverage. The same procedure resulted in OFV values of 0.45 for organs requiring progressively less aggressive dose reduction.

**Fig. 1:**
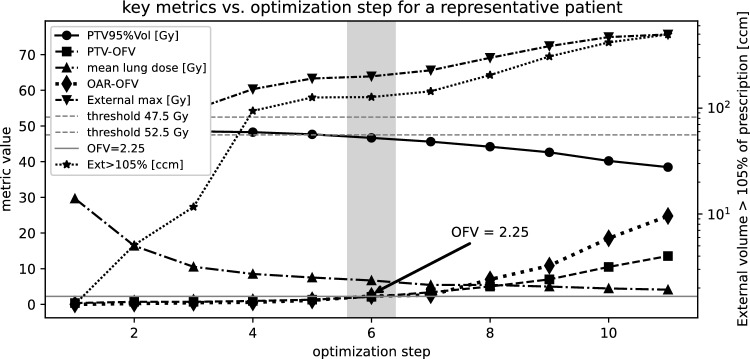
Shown are the key metrics for each optimization step: PTV coverage (PTV95 %Vol), PTV-OFV, external maximum dose, external volume> 105 % of the prescription dose, OAR-OFV, and mean OAR dose. The plot illustrates that for an OAR-OFV value of approximately 2.25, further sparing of the OAR dose is no longer feasible without compromising PTV dose coverage (PTV95 %Vol). Further reduction of the mean lung dose also leads to an increase in the maximum dose of the treatment plan.

### Combining OFV with linear functions

The optimization process was guided by clinical objectives defined for individual organs at risk and by established characteristics of the underlying mathematical models, including dose fall-off behavior, equivalent uniform dose (EUD), and dose–volume histogram (DVH) metrics, with particular consideration of serial and parallel organ properties. All relevant target volumes and organs at risk were contoured prior to planning as part of the standard clinical workflow.

Treatment planning was performed using a standardized technical setup identical for both clinical reference plans and automated plans, as detailed in Section "Patient selection and technical equipment used".

Automation was introduced exclusively at the optimization stage. The proposed approach was empirically refined by analyzing the behavior of the OFV during the optimization process in RayStation 11B. Based on these observations, MaxEUD constraints were iteratively adapted in response to the observed OFVs, allowing systematic exploration of organ-at-risk sparing while maintaining target coverage.

We observed that the OFV for PTV coverage typically exceeds 1, while acceptable sparing of OARs can often be achieved with OFVs in the range of 0.1 to 1. However, optimal treatment of breast cancer requires maximal sparing of the heart, ipsilateral lung, and contralateral breast to achieve organ doses comparable to those of conventional 3DCRT. For this purpose - specifically using our individual weighting values for optimization - an OFV greater than 1 is necessary for these critical OARs, while maintaining satisfactory PTV coverage. As previously mentioned, our approach relies on accessing and adjusting the OFV via Python scripting (included within RayStation).

**Fig. 2:**
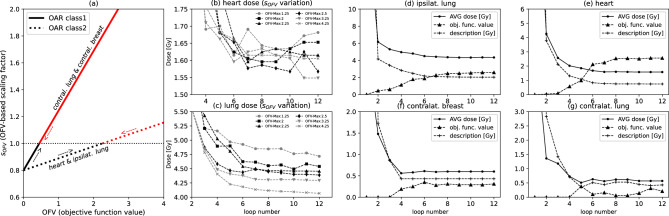
(**a**): Basic illustration of the combination of the OFV with two linear functions as used in our approach. After each optimization step (including 40 iterations) the OFV of every organ at risk is read in by the Python script and the MaxEUD setting is either increased or further decreased (current average dose is multiplied by $s_{OFV}$). The red colored parts denote the regions where the OFV has already reached a certain limit and the appropriate MaxEUD setting of the individual organ (either class1 or 2) is softened before the next optimization is started. The black parts show the OFV range where still further organ dose reduction is possible. The optimization process is fully automatic and shown in panels (**d**)-(**g**) with the help of a representative patient. It is demonstrated that after only a few optimizations a saturation is reached using our approach. Until about 5–6 optimizations the organ dose is significantly reduced and further organ dose reduction can then only be reached for class2 organs (heart and ipsilateral lung) allowing for a high OFV>2. The description (OAR MaxEUD setting) is dynamically decreased or increased (indicated by arrows in (**a**)) before the next optimization loop to avoid a high OFV that would lead to an increased underdosage of the PTV. This dynamic adaptation is evident in panel (**e**) for the contralateral lung.

Two linear functions (Fig. 2a) determine how the MaxEUD setting for each organ is adjusted, either decreased or increased, prior to the next optimization step. The optimization is fully automated and proceeds through multiple phases. In Phase 1, the MaxEUD setting for each OAR is set equal to the prescribed dose. After this initial optimization, the resulting mean organ doses are used to define MaxEUD settings for Phase 2. Specifically, for class 1 organs (contralateral lung, contralateral breast), the MaxEUD is set to 50 % of the Phase 1 mean dose. For class 2 organs (ipsilateral lung and heart), it is set to 20 % of the mean dose of Phase 1. In Phase 3, the linear equations $s_{\text {OFV}} = 0.44 \cdot \text {OFV}_{\text {OAR}} + 0.8$ (class1 organ) and $s_{\text {OFV}} = 0.089 \cdot \text {OFV}_{\text {OAR}} + 0.8$ (class2 organ) are used to obtain the MaxEUD settings of organs via $\text {MaxEUD}_{\text {OAR, PHASE}_{n}} = s_{\text {OFV}} \cdot \text {MaxEUD}_{\text {PHASE}_{n-1}}$ for every further step. This is illustrated in Fig. 2a. For example, the MaxEUD of a class 1 OAR with OFV > 0.45 is increased ($\text {MaxEUD}_{n+1} \ge \text {MaxEUD}_{n}$), as further lowering it would worsen the OFV and compromise sparing of more critical class 2 organs. Conversely, the MaxEUD for class 2 organs is reduced iteratively until an OFV of approximately 2.25 is reached. This upper OFV limit avoids excessive sparing that could result in underdosage of the PTV (Fig. 1). Figure 2d-g also visualizes how the OFV, MaxEUD setting, and average dose evolve over 12 optimization loops for a representative patient. Based on our experience, 6–8 optimization loops are generally required to achieve satisfactory results.

Following completion of the automated optimization, PTV coverage was occasionally insufficient. Therefore, an auxiliary structure representing underdosed regions was generated by creating a Boolean intersection between the PTV and the 95 % isodose volume. This structure was incorporated into one or two additional optimization iterations to restore clinically acceptable target coverage. When non-tangential fluence increased in cranial or caudal PTV regions because the lung did not span the full target height, a surrogate contour approximating lung geometry was introduced and optimized using identical OFV settings to maintain tangential planning behaviour.

However, due to differences in mathematical optimization settings, contouring standards and internal dose constraints across institutions, further adjustments to the linear functions–particularly the slope (m) and intercept (n)–may be necessary when applying our method in other settings.

The behavior of the empirically derived linear functions depends on the geometric relationship between the PTV and adjacent organs at risk, including the ipsilateral lung, heart, and contralateral breast. In the present implementation, MaxEUD constraints are adapted exclusively based on the observed OFVs of the OARs, without explicitly incorporating the OFV of the PTV. As PTV coverage is preserved throughout optimization, its influence is implicitly accounted for.

To assess the robustness of the empirically derived maximum OFV, we evaluated the automated optimization procedure across six representative OFV thresholds: 1.25, 2.0, 2.25, 2.5, 3.25 and 4.25. These thresholds were chosen to cover the approximate range in which the PTV V95 % criterion begins to be violated (Fig. 1), reflecting realistic variations a user might select in practice. For each OFV threshold, the resulting dose distributions of ipsilateral lung and heart were analyzed for a representative patient (Fig. 2b,c). The results demonstrate that the optimization outcome is highly robust to moderate variations in the maximum OFV value. These results confirm that exact parameter tuning is not required; selecting an OFV threshold approximately in the range where PTV coverage starts to decline is sufficient to obtain reliable and clinically acceptable dose distributions. This demonstrates that the empirically derived maximum OFV approach is straightforward, reproducible, and robust across moderate parameter variations.

## Results

**Fig. 3:**
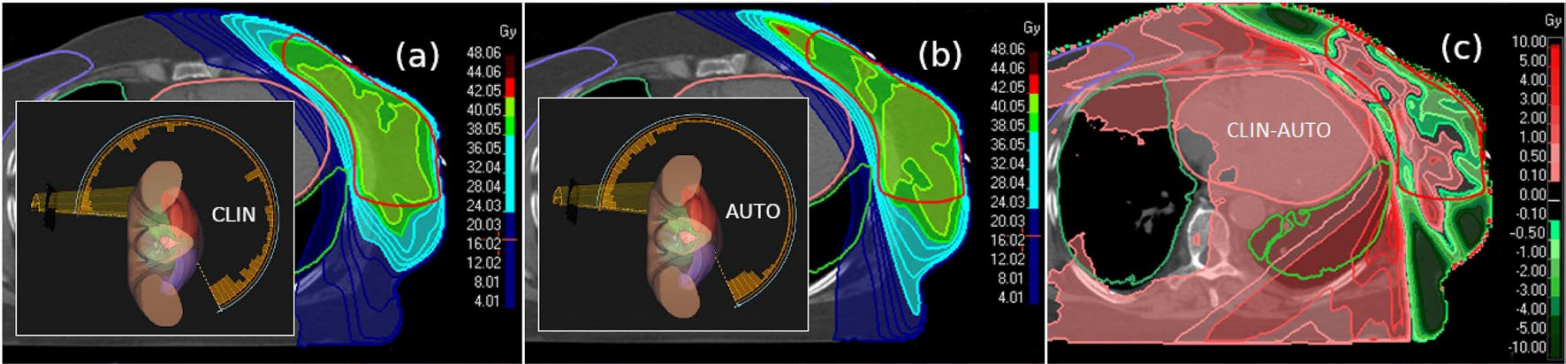
Screenshots of a representative patient showing the qualitative and quantitative differences in dose rate delivery between the two plan types (**a**): clinical-plan and (**b**): automatic-plan. Dose of the clinical plan is less tangential with the consequence of increased low dose burden to the whole patient body due to the impact of segmentation from ventro-lateral direction (as demonstrated by the two smaller plots showing the arc and angle specific monitor units). It can be seen that the treatment plan obtained via our approach (**b**) is treating the patient mainly from tangential directions whereas the clinical plan (**a**) delivers dose also from ventro-lateral directions which should be avoided. The lower panel (**c**) shows the dose difference between both plans demonstrating the increased low-dose volume in the contralateral patient side for the originally delivered clinical plans.

To visualize the qualitative differences between the clinical plan and the automatically generated plan, we created screenshots of the dose distribution for a representative patient, as shown in Fig. 3. It can be observed that the automatic plan produces a dose distribution that closely resembles a tangential beam arrangement, thereby increasing the prescribed isodose volume (PIV). However, this results in a reduced low-dose burden to the patient on the contralateral side, as demonstrated by the difference plots in Fig. 3c. The quantitative impact of these findings on organ-at-risk doses is analyzed in the following sections. For plan evaluation, various parameters were assessed, including the conformity index (*CI*), homogeneity index (*HI*), prescribed isodose volume (PIV), dose probability density, standard statistical measures (minimum, maximum, mean, standard deviation), and dose-volume metrics such as V_5Gy_, V_10Gy_, and V_20Gy_. The *CI* was calculated according to the definition proposed by^61^: $CI = \frac{PIV_{PTV}}{PTV_{VOL}} \times \frac{PIV_{PTV}}{PIV_{EXT}}$ where $PIV_{PTV}$ is the portion of the planning target volume (PTV) covered by the 95 % isodose, $PTV_{VOL}$ is the total volume of the PTV, and $PIV_{EXT}$ is the total patient volume receiving more than 95 % of the prescribed dose. *CI* values range from 0 to 1, with higher values indicating better conformity. The homogeneity index (*HI*) was calculated as $HI = \frac{D_5}{D_{95}}$, following^62^. Here, D_5_ and D_95_ refer to the doses received by 5 % and 95 % of the PTV, respectively. A higher *HI* indicates lower dose uniformity within the PTV. Although a wide variety of quality indices have been proposed in the literature^63^, it is widely recognized that no single metric provides a comprehensive assessment of plan quality. Therefore, in this study, we focused on CI and HI in combination with additional parameters: the prescribed isodose volume, dose-volume metrics, dose probability density, statistical significance (p-values), and dose distribution plots. This approach offers both quantitative and qualitative insights into the differences between the clinical and automatically generated treatment plans evaluated in this study.

### Target coverage, conformity, and peripheral dose bath

Target coverage and dose homogeneity were evaluated using the *HI*, *CI*, and key volumetric parameters (Table 1). Both clinical and automated plans achieved excellent coverage, with a$V_{95\%}$of $97.55\,\%$ and $96.98\,\%$ ($p = 0.212$), respectively, confirming that the automated approach reliably maintains oncological safety. Dose homogeneity remained nearly identical, with *HI* values of $1.068 \pm 0.018$ (Clinical) versus $1.071 \pm 0.014$ (Auto-Opt) with $p = 0.456$. While hot spots ($V_{105\%}$) were slightly reduced in the automated plans ($0.95\,\%$ vs. $0.87\,\%$), this difference was not statistically significant ($p = 0.813$).

A significant divergence was observed regarding the Paddick Conformity Index, where automated plans showed lower values compared to clinical plans ($0.829 \pm 0.056$ vs. $0.76 \pm 0.055$, $p < 0.001$). However, this reduction in *CI* does not indicate a loss of planning quality, but rather a strategic shift in dose distribution. The automated algorithm favors a more tangential beam arrangement, which intentionally expands the prescribed isodose volume (*PIV*) into non-critical peripheral areas to maximize the sparing of central OARs.

This strategy is quantitatively supported by the significant reduction of the peripheral dose bath within the External volume (Table 1). The automated plans reduced the low-dose exposure substantially: the $V_{1Gy}$ decreased from $11044 \pm 2878\,\text {cm}^3$ to $8225 \pm 1955\,\text {cm}^3$ ($p < 0.001$) and the $V_{5Gy}$ from $4312 \pm 1402\,\text {cm}^3$ to $3508 \pm 1164\,\text {cm}^3$ ($p < 0.001$). By effectively ’pushing’ the dose out of the patient’s body in tangential directions, the algorithm significantly reduces the integral dose and the ’low-dose bath’ associated with conventional VMAT planning. This global sparing of healthy tissue potentially reduces the risk of secondary malignancies while maintaining clinically acceptable target coverage^39^. reported an increase in monitor units of approximately $36\,\%$ when using in-house scripting for planning with Pinnacle³. Our automated approach significantly improved delivery efficiency, as evidenced by a substantial reduction in Monitor Units (MU) from $620 \pm 64$ to $515 \pm 54$ ($p < 0.001$). This decrease in MU suggests a lower modulation complexity, which contributes to the reduction of integral dose and peripheral scatter. This is attributable to a lower number of small beam segments being delivered from ventrolateral directions, which reduces the total MU requirement for the automatically generated plans. The beam-on treatment times were comparable between the two plan types, with an average difference of less than 20 seconds (automatic: $242 \pm 29~\textrm{s}$; clinical: $259 \pm 44~\textrm{s}$).

The present work focuses on the technical feasibility and the resulting plan quality of the automated workflow. Although planning time was not explicitly recorded, the process demonstrated high stability, consistently reaching convergence within 6–8 optimization loops (as illustrated in Fig. 2). The primary benefit of the proposed method lies in the elimination of inter-planner variability and the achievement of an objective, optimized dose distribution that has the potential to exceed manual standards.

**Table 1:** Dosimetric comparison of target coverage, conformity and peripheral dose bath between clinical and automated plans. ”*PTV opt*” denotes the physical optimization volume used for inverse planning.

Parameter	Clinical	Auto-Opt	Unit	*p*-value
*Monitor* *Units* :	620 ± 64	515 ± 54	cm$^3$	< 0.001
PTV opt:				
Mean Dose	50.33 ± 0.18	50.27 ± 0.15	Gy	0.011
$V_{95\%}$	97.55 ± 1.713	96.98 ± 1.189	%	0.212
$V_{105\%}$	0.95 ± 1.42	0.87 ± 0.62	%	0.813
HI	1.068 ± 0.018	1.071 ± 0.014		0.456
CI (Paddick)	0.829 ± 0.056	0.76 ± 0.055		< 0.001
External:				
$V_{1Gy}$	11044 ± 2878	8225 ± 1955	cm$^3$	< 0.001
$V_{5Gy}$	4312 ± 1402	3508 ± 1164	cm$^3$	< 0.001
$V_{10Gy}$	3090 ± 1079	2843 ± 1012	cm$^3$	< 0.001
$V_{20Gy}$	2333 ± 878	2294 ± 878	cm$^3$	0.159

### Dose reduction in organs at risk

The dosimetric comparison between clinical and automated VMAT plans regarding the OAR doses (Table 2) reveals a significant reduction in the dose burden for all evaluated OARs when using the OFV-guided approach.

Regarding the cardiac load, the mean heart dose (MHD) was significantly reduced from $3.09 \pm 1.06$ Gy to $2.21 \pm 0.69$ Gy ($p < 0.001$). This improvement is particularly evident in the low-dose range, where the $V_{2Gy}$ of the heart decreased by approximately $42\,\%$ (from $37.60\,\%$ to $21.71~\mathrm{\%}$). While high-dose parameters like $D_{1\%}$ and $V_{20Gy}$ did not show statistically significant differences, the reduction in mean and low dose is clinically relevant for long-term cardiovascular health.

The automated approach also demonstrated superior performance in sparing the lungs and the contralateral breast. For the ipsilateral lung, the mean dose was lowered from 8.49 Gy to 7.23 Gy ($p < 0.001$), and for the contralateral lung, the dose was nearly halved (1.52 Gy vs. 0.83 Gy; $p < 0.001$). The most striking difference was observed in the $V_{2\text {Gy}}$ of the contralateral lung, which dropped from $21.80~\mathrm{\%}$ to $2.40~\mathrm{\%}$ ($p < 0.001$). Similarly, the mean dose to the contralateral breast was reduced from 2.29 Gy to 1.43 Gy ($p < 0.001$). These results indicate that the OFV-guided iterative update of MaxEUD constraints successfully identifies the physical limits of dose sparing for each individual anatomy. This systematic dose reduction is further visualized in the population-based dose-volume histograms (Fig. 5). The DVH curves clearly demonstrate that the automated approach (dashed lines) achieves a consistent downward shift across the entire low- and mid-dose range for all critical OARs compared to clinical plans (solid lines), while the narrow shaded areas confirm a high degree of inter-patient plan consistency.

Furthermore, the spinal cord showed significant improvements in mean dose and $D_{1\%}$, further highlighting the algorithm’s ability to minimize the ”low-dose bath” often associated with VMAT. As noted in the overall study context, these OAR sparing effects were achieved alongside a $16.9~\mathrm{\%}$ reduction in monitor units (MU). This suggests that the automated plans are not only dosimetrically superior but also more efficient in terms of delivery, potentially reducing the risk of secondary malignancies by lowering the overall integral dose.

**Table 2:** Dosimetric comparison of OAR sparing between clinical and automated plans for heart, contralateral breast, ipsilateral/contralateral lung and spinal cord.

Parameter	Clinical (Mean ± SD)	Automatic (Mean ± SD)	Unit	*p*-value
Heart				
Mean Dose (L/R)	3.09 ± 1.06/1.88 ± 1.06	2.21 ± 0.69/1.17 ± 0.25	Gy	$<0.001 / <0.001$
Mean Dose (all)	2.67 ± 1.20	1.85 ± 0.76	Gy	$< 0.001$
$D_{1\%}$	11.05 ± 8.76	9.38 ± 8.88	Gy	0.150
$V_{2Gy} / V_{5Gy}$	37.60 ± 32.41/7.40 ± 8.10	21.71 ± 18.20/3.12 ± 3.10	%	$<0.001 / 0.007$
$V_{10Gy} / V_{20Gy}$	2.16 ± 2.73/0.88 ± 1.04	1.47 ± 1.64/0.78 ± 0.81	%	0.044/0.420
Lungs (Ipsilateral/Contralateral)				
Mean Dose	8.49 ± 1.06/1.52 ± 0.50	7.23 ± 1.06/0.83 ± 0.17	Gy	$< 0.001 / < 0.001$
$D_{1\%}$	45.68 ± 3.16/4.80 ± 1.67	46.10 ± 2.85/2.27 ± 0.72	Gy	$0.097 / < 0.001$
$V_{2Gy}$	75.80 ± 10.10/21.80 ± 17.10	55.70 ± 6.30/2.40 ± 3.50	%	$< 0.001 / < 0.001$
$V_{5Gy}$	43.40 ± 10.30/1.52 ± 1.80	30.40 ± 3.60/0.03 ± 0.11	%	$< 0.001 / 0.002$
$V_{10Gy}$	30.40 ± 3.60/0.04 ± 0.12	19.10 ± 3.10/0.00 ± 0.00	%	$< 0.001 / 0.141$
$V_{20Gy}$ (ipsilateral only)	12.70 ± 3.01	12.00 ± 2.80	%	0.287
Contralat. Breast				
Mean/$D_{1\%}$	2.29 ± 0.96/9.88 ± 4.87	1.43 ± 0.56/8.62 ± 6.62	Gy	$< 0.001 / 0.245$
$V_{2Gy} / V_{5Gy}$	35.80 ± 23.70/8.30 ± 8.13	14.50 ± 9.20/2.90 ± 3.40	%	$< 0.001 / 0.005$
$V_{10Gy} / V_{20Gy}$	1.34 ± 1.70/0.16 ± 0.35	1.02 ± 1.60/0.25 ± 0.54	%	0.362/0.446
Spinal Cord				
Mean/$D_{1\%}$	1.07 ± 0.54/2.84 ± 1.75	0.75 ± 0.27/1.88 ± 0.86	Gy	0.009/0.019
$V_{2Gy} / V_{5Gy}$	15.28 ± 18.33/0.87 ± 2.36	3.91 ± 7.78/0.00 ± 0.00	%	0.016/0.124

**Fig. 4:**
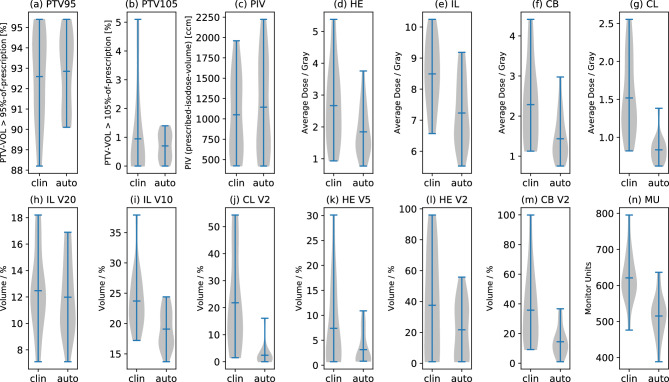
Violin plots showing the probability density distribution regarding (**a**) the volume of PTV greater than 95 % of the prescription, (**b**) the volume of PTV greater than 105 % of the prescription and (**c**) the volume of the patient receiving 95 % of the prescription (PIV: prescribed isodose volume). Panels (**d**)-(**g**): Violin plots (probability density distribution with min, max and average dose markers) for the organs at risk. Panels (**h**)-(**m**): Violin plots for dose-volume relations (probability density distribution with min, max and average dose markers) for the organs at risk and panel (**n**) showing the monitor units. (HE: heart, IL: ipsilateral lung, CB: contralateral breast, CL: contralateral lung, MU: monitor units).

Importantly, our approach did not result in increased high-dose exposure to any organ at risk, as confirmed by the probability density distributions presented in Fig. 4. Beyond the reduction of absolute dose values, Fig. 4 further illustrates a significant decrease in inter-patient dose variability within the automatically generated plans. The narrower shapes of the violin plots for nearly all parameters, but especially for PTV dose coverage, heart, contralateral breast, contralateral lung and low dose volumes (Fig. 4a,d,f,g,j,k,m) indicate a more consistent and predictable planning outcome compared to the clinical cohort.

**Fig. 5:**
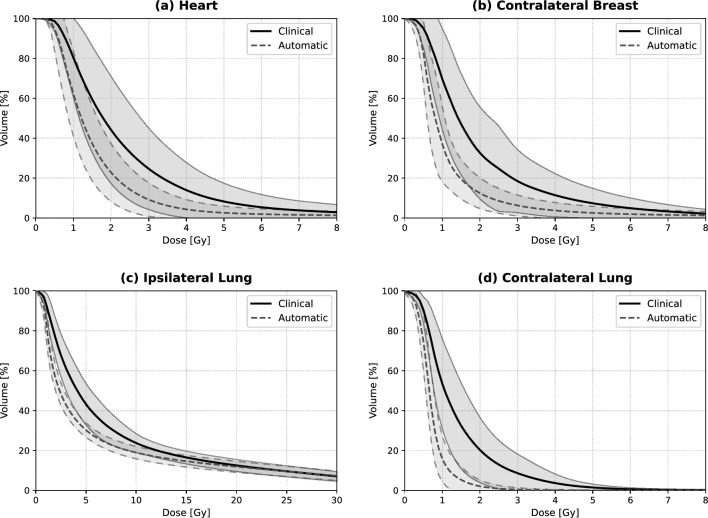
Comparison of mean Dose-Volume Histograms (DVH) for clinical plans (solid lines) versus automated optimization (dashed lines) across 20 patients. Shaded areas represent the standard deviation (SD). For clarity, SD boundaries are shown as solid lines for clinical plans and widely dashed lines for automated plans.

The downward shift of the entire probability density in the automated plans demonstrates that OAR sparing is not merely achieved for a subset of patients but is a systematic improvement. Furthermore, the reduction in the spread between minimum and maximum values (min/max markers) suggests that the OFV-guided optimization effectively minimizes dose outliers that can occur in manual planning due to time constraints or varying planner experience.

To further investigate whether this systematic benefit remains consistent across diverse patient anatomies, the relationship between PTV volume and dose reduction ($\Delta$ Dose) was examined. Pearson’s correlation analysis confirmed that dose savings were independent of target size across the entire spectrum of PTV volumes ($1039 \pm 495$ $\text {cm}^3$). Specifically, no significant correlation was found for the heart ($r = 0.07$, $p = 0.763$), ipsilateral lung ($r = -0.11$, $p = 0.637$), contralateral lung ($r = 0.12$, $p = 0.627$), or contralateral breast ($r = -0.04$, $p = 0.865$). This lack of significant correlation ($p> 0.05$ for all OARs) demonstrates that the automated approach provides a consistent and predictable benefit regardless of breast volume. Consequently, the observed dosimetric improvements can be considered a systemic advantage of the optimization process rather than a result of anatomical variability.

### Robustness analysis in atypical anatomy (funnel chest case)

**Fig. 6:**
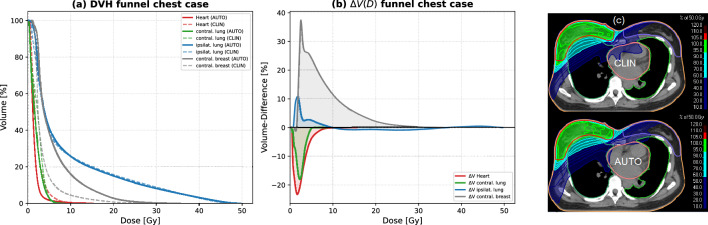
Dosimetric comparison for the funnel chest case. (**a**) Cumulative dose-volume histograms (DVHs) for the heart, ipsilateral lung, contralateral lung, and contralateral breast, comparing the clinical reference plan (CLIN) and the automated OFV-guided optimized plan (AUTO). (**b**) Dose-volume difference curves $\Delta V(D) = V_{\textrm{AUTO}}(D) - V_{\textrm{CLIN}}(D)$, illustrating volume reductions for the heart and contralateral lung and increased dose exposure for the contralateral breast in the automated plan, while ipsilateral lung dose remains largely unchanged. (**c**) Axial dose distributions at the level of maximum cardiac proximity for the clinical (top) and automated (bottom) plans, demonstrating a more tangential beam geometry in the automated optimization, resulting in reduced cardiac and contralateral lung exposure and increased incidental dose to the contralateral breast.

Funnel chest (pectus excavatum) represents a pronounced anatomical deviation that can substantially influence beam geometry and dose trade-offs in left-sided breast radiotherapy. To assess the robustness and characteristic behavior of the proposed automated OFV-guided optimization framework under such conditions, an additional case-based analysis was performed in a patient with funnel chest anatomy (Haller index 3.6). This patient was not included in the main cohort and was selected solely for illustrative purposes to demonstrate the limitations of the method in geometrically complex cases. The automated optimization was applied using identical objective functions, weighting factors, and planning constraints as used for the cohort analysis, without anatomy-specific manual adaptation. As summarized in Table 3, the automated plan achieved a pronounced reduction in cardiac dose, with the mean heart dose decreasing from $2.17~\textrm{Gy}$ to $1.46~\textrm{Gy}$, accompanied by lower low-dose exposure ($V5~\textrm{Gy}$: $5.64~\mathrm{\%}$ to 1.76 %; $V10~\textrm{Gy}$: $0.43~\mathrm{\%}$ to $0.29~\mathrm{\%}$). A reduction in mean dose to the contralateral lung was observed as well ($2.29~\textrm{Gy}$ to $1.89~\textrm{Gy}$). Dose metrics for the ipsilateral lung remained largely unchanged, with comparable mean dose and low-dose exposure. In contrast, the automated plan resulted in a marked increase in mean dose to the contralateral breast ($3.53~\textrm{Gy}$ to $5.94~\textrm{Gy}$). This trade-off is clearly illustrated by the dose-volume histograms shown in Fig. 6a and by the corresponding dose-volume difference curves ($\Delta V(D)$, Fig. 6b).

**Table 3:** Comparison of clinical and automatic treatment plans for the funnel chest test case.

Structure	Metric	Clinical	Automatic
Heart	Mean [Gy]	2.17	1.46
	V_5Gy_ [%]	5.64	1.76
	V_10Gy_ [%]	0.43	0.29
Ipsilateral Lung	Mean [Gy]	8.91	9.04
	V_10Gy_ [%]	25.8	25.74
	V_20Gy_ [%]	15.63	14.96
Contralateral Lung	Mean [Gy]	2.29	1.89
Contralateral Breast	Mean [Gy]	3.53	5.94
	V_5Gy_ [%]	14.49	38.66
	V_10Gy_ [%]	4.48	16.03

The axial dose distributions depicted in Fig. 6c further support this interpretation. Compared to the clinical plan, the automated optimization favors more tangential beam configurations, effectively reducing cardiac and contralateral lung exposure. In the presence of severe funnel chest anatomy, such tangential geometries may increase incidental dose to the contralateral breast due to altered thoracic geometry and reduced mediastinal separation. This single-case analysis demonstrates that the automated OFV-guided optimization framework remains technically robust and effective in reducing cardiac dose even in extreme anatomical configurations. At the same time, it highlights a relevant limitation: in patients with pronounced funnel chest anatomy, improved cardiac sparing may be associated with increased contralateral breast dose. These findings emphasize the importance of institution-specific dose prioritization and suggest that additional anatomy-aware constraints or adaptive objective weighting strategies may further improve performance in such cases. This analysis is not intended for statistical inference but serves to illustrate characteristic dose trade-offs in atypical anatomy.

### Comparing OFV-guided results with literature

**Fig. 7:**
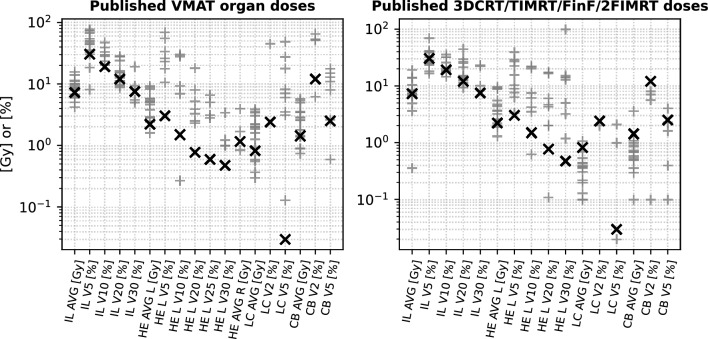
Review of published OAR doses for VMAT (left panel) and 3DCRT treatment plans (right panel). The ’x’ denotes the VMAT plan data obtained using our OFV-approach. Note that the y-axis is displayed on a logarithmic scale to accommodate the wide range of dose–volume values reported in the literature; corresponding numerical values for the OFV-based plans are provided in Tables 4, 5 and 6.

As the dosimetric comparison in this study is primarily based on two plan types generated within our own department, it is essential to place our findings in the context of published data to assess the full potential of our OFV-guided approach. The upper panel of Fig. 7 illustrates the dosimetric differences in terms of average organ doses and dose-volume relationships for the VMAT treatment plans analyzed. Overall, OFV-guided plans yielded lower organ doses compared to those reported in the literature, particularly with regard to (i) the mean heart dose and its associated dose-volume parameters, (ii) the mean ipsilateral lung dose, (iii) the mean contralateral breast dose, and (iv) the mean contralateral lung dose. Most notably, significant reductions were observed in heart dose as well as in the V_2Gy_ and V_5Gy_ values across all evaluated organs. When comparing OFV-guided VMAT data with published results for 3DCRT, the ipsilateral lung doses were found to be similar. However, the OFV-guided plans showed a tendency for improved high-dose-volume sparing. Surprisingly, our approach also demonstrated superior heart sparing compared to 3DCRT, a finding that challenges the common assumption that 3DCRT is always more protective in this regard. On the other hand, mean doses to the contralateral breast and contralateral lung were somewhat higher with VMAT–a logical trade-off considering the improved heart sparing and overall reduction in high-dose volumes achieved by the technique.

After evaluating our results in the context of published data, we identify an important aspect of current research: the ongoing investigation into secondary cancer risk when comparing 3DCRT and VMAT. For instance^2^, concluded that 3DCRT may offer 34 % (linear model) to 50 % (linear-exponential and plateau models) lower secondary cancer risk compared to VMAT. Their reported mean doses for 3DCRT and VMAT align with values commonly found in the literature. However, in comparison to our OFV-guided VMAT data, their plans yielded significantly higher mean doses: contralateral breast dose: +0.35 Gy (+24 %), ipsilateral lung dose: +2.7 Gy (+37 %) and contralateral lung dose: +1.2 Gy (+140 %). We acknowledge that their VMAT plans likely represented the best achievable results at the time. Nonetheless, with the advent of modern linear accelerators featuring dynamic gantry speed and dose rate modulation, the mathematical and mechanical degrees of freedom have expanded considerably–enabling substantially improved sparing of organs at risk. A further example is the study by^4^, which also examined secondary cancer risk in the context of VMAT versus 3DCRT. Their reported V_2Gy_ for the contralateral breast was 65 %, compared to just 12 % in our approach. For the contralateral lung, they reported a V_2Gy_ of 45 %, while our method achieved only 2.4 %. Dose-volume differences for the ipsilateral lung were also substantial (see Fig. 7 and Table A1 for details).

**Table 4:** OAR dose data from different publications for VMAT (IL=ipsilateral lung; LC=contralateral lung; HE=heart; CB=contralateral breast; L=left-sided breast cancer; R=right-sided breast cancer): Additional information: for^64^ we took Breast and Chest wall patients and scaled the data from originally 40 Gy to 50 Gy, data of^65^ is based on rather untypical geometry compared to a real patient leading to a very low ipsilateral lung dose, from^66^ we took VMAT-FB data, from^18^ we took the VMAT-2Arc data, from^67^we took the C-VMAT data^68^, average doses were scaled to 50 Gy from 42.7 Gy, from^21^ we took 4 A H-VMAT data, from^11^ we took VMAT-FB data, from^27^ we took VMAT-FB data, from^69^ we took the Elekta VMAT data, from^4^ we took the data directly from the figures, data from^70^ was scaled from 40,05 to 50Gy in the Table. **OAR dose data from different publications for 3DCRT or equivalent techniques (T-IMRT, TVMAT):**(IL=ipsilateral lung; LC=contralateral lung; HE=heart; CB=contralateral breast; L=left-sided breast cancer; R=right-sided breast cancer). Additional information^68^: average doses were scaled to 50 Gy from 42.7 Gy, from^11^ we took 3DCRT FB data, from^27^ we took 3DCRT FB data, from^15^ we took the field-in-field (FIF, 3DCRT) and tangential intensity-modulated radiotherapy (T-IMRT, a hybrid technique between 3DCRT and IMRT) data, from^18^ took the two-field IMRT (2FIMRT) data, data of^65^ is based on rather untypical geometry compared to a real patient leading to a very low ipsilateral lung dose, from^4^ we took the data directly from the figures, from^67^ we took FinF data, cumulative dose in^3^ was 50.4 Gy.

VMAT					3DCRT				
IL AVG	HE AVG	CL AVG	CB AVG	Publication	IL AVG	HE AVG	CL AVG	CB AVG	Publication
[Gy]	[Gy]	[Gy]	[Gy]		[Gy]	[Gy]	[Gy]	[Gy]	
7,23	2,21	0,83	1,43	Automatic	7,23	2,21	0,83	1,43	Automatic
9,9	-	1,99	1,78	^2^	6,7	4,7	0,38	3,59	^68^
5,95	-	2,18	5,31	^65^	19	9	0,4	0,7	^11^
10,1	4,6	-	-	^15^	13,9	9,54	0,36	0,69	^71^
5	-	0,3	0,9	^18^	3,6	2,54	-	-	^27^
8,1	2,4	0,37	0,75	^70^	6,6	1,3	0,1	0,1	^69^
8,7	-	1,6	2,6	^67^	8,2	3,2	-	-	^15^
15,7	8,2	-	5,7	^66^	6,8	2,2	-	-	^15^
9,18	5,1	0,56	0	^64^	4,9	-	0,2	0,5	^18^
11,3	5,37	3,85	3,45	^20^	0,36	-	0,13	0,36	^65^
7,93	8,79	3,52	4,93	^68^	13,85	8,8	1,08	0,92	^72^
8,47	5,72	1,27	1,4	^21^	8,14	5,5	0,22	0,74	^21^
14	5,8	3,4	2,8	^11^	7,21	4,39	0,45	0,55	^22^
8,4	4,97	1,85	3,56	^71^	8,1	2,6	0,1	0,3	^73^
4,18	2,91	-	-	^27^	8,5	-	0,38	1,1	^3^
6,7	1,6	0,6	0,9	^69^	-	-	-	-	^4^
-	-	-	-	^4^	10,4	9,1	0,7	1	^67^
9,9	-	1,99	1,78	^2^	7,4	-	0,44	0,51	^2^
	5,15	-	-	^74^		3,7	-	-	^74^
11,08	9,24	3,03	2,56	^22^	6,7	3,6	-	-	^75^
8,5	3,8	-	-	^75^	7,3	2,01	0,54	0,86	^76^
6,23	2,47	0,93	2,87	^76^	7,24	1,88	0,31	0,59	^77^
8,61	2,79	2,22	3,44	^77^	-	-	-	-

**Table 5:** VMAT dose-volume-parameter from different publications. For further details see caption of Table 4.

IL V5	IL V10	IL V20	IL V30	HE V5	HE V10	HE V20	HE V25	HE V30	CL V2	CL V5	CB V2	CB V5	**Publication**
[%]	[%]	[%]	[%]	[%]	[%]	[%]	[%]	[%]	[%]	[%]	[%]	[%]	
30.4	19.1	12	7.5	3.03	1.5	0.77	0.6	0.48	2.4	0.03	12	2.5	OFV
50.3	29.9	16.4	-	26.1	6.9	2.5	-	-	-	-	-	-	^15^
18.4	-	9.2	-	-	-	-	-	-	-	0	-	0.6	^18^
8.1	-	-	-	-	-	-	-	-	-	-	-	-	^70^
35.5	23.4	15.3	5.5	-	-	-	5	3.4	-	-	53	-	^67^
77.2	47.1	28.2	-	-	-	-	3.1	-	-	48.5	-	-	^66^
73.1	-	15.3	-	-	-	2.3	-	-	-	27.5	-	-	^20^
43.8	-	11.03	-	23	-	-	6.56	-	-	0.13	-	2.62	^21^
67.5	-	27.9	-	33.6	-	3.9	-	1	-	17.8	51.3	12.9	^11^
32.5	20.5	13.4	9.33	17.7	9.23	5.22	-	1.24	-	3.1	-	-	^71^
30.2	20.3	12.6	-	-	-	-	-	-	-	-	6.2	-	^69^
45	33	24	19	55	30.0	18.0	-	1	45	7	65	15	^4^
-	-	9.65	-	-	-	3.28	-	-	-	-	-	-	^74^
73	39	13.7	-	69	27.9	8	-	-	-	6	-	7.99	^22^
48.6	-	13.3	-	-	-	-	-	-	-	3.5	-	10.9	^75^
-	-	8.72	-	-	-	-	2.79	-	-	-	-	-	^76^
54.14	28.36	11.21	4.96	10.56	0.27	0	-	0	-	8.16	-	17.6	^77^

**Table 6:** 3DCRT (or equivalent) dose-volume-parameter from different publications. For further details see caption of Table 4.

IL V5	IL V10	IL V20	IL V30	HE V5	HE V10	HE V20	HE V30	CL V2	CL V5	CB V2	CB V5	Publication
[%]	[%]	[%]	[%]	[%]	[%]	[%]	[%]	[%]	[%]	[%]	[%]	
-	-	-	-	-	-	-	-	-	-	-	-	^68^
69.4	-	44.2	-	39.1	-	16.6	12.8	-	0	5.6	1.6	^11^
38.2	32.11	26.7	23.1	26.95	21.72	17.25	14.28	-	0.02	-	-	^71^
25.9	17.7	14.2	-	-	-	-	-	-	-	0.1	-	^69^
24.6	19.1	15	-	8.9	6.1	4.3	3.2	-	-	-	-	^15^
23.4	17.7	12.9	-	6.3	3.5	2	1.2	-	-	-	-	^15^
16.6	-	9.3	-	-	-	-	-	-	0	-	0.1	^18^
41.67	35.6	29.5	-	15.6	20.11	-	-	-	2.08	-	-	^72^
40.3	-	11.2	-	22.4	-	-	-	-	0	-	2.25	^21^
18.18	14.51	12.51	-	10.3	7.5	6	5	-	-	-	0	^22^
28.1	20.3	14.8	-	7.6	4.2	2	-	-	-	-	-	^73^
40	32	25	22	29	22	17	14	2	1	8	4	^4^
32	25.7	21.4	-	-	-	-	15.2	-	-	7	-	^67^
-	-	11.4	-	-	-	4.65	-	-	-	-	-	^74^
24.8	-	14.1	-	0	-	-	-	-	-	-	0.4	^75^
-	-	13.35	-	-	-	-	-	-	-	-	-	^76^
28.04	18.3	12.49	10.05	3.32	0.63	0.11	-	-	0	-	0	^77^

## Discussion

Our findings demonstrate that OFV-guided automatic VMAT planning enables substantial sparing of OARs without compromising target coverage. Significant dose reductions (p<0.001) were observed for the heart, ipsilateral and contralateral lungs, and contralateral breast, alongside a significant reduction in monitor units (p<0.001). These results align with the auto-planning literature reporting comparable target coverage and improved reproducibility relative to manual planning^32–36^. In contrast to workflows that show increased monitor units or elevated low-dose exposure^39,41,42,78–80^, the proposed approach achieved OAR reductions without these trade-offs.

Although workflow duration was not assessed, the fully automated optimization removes iterative manual tuning and is expected to reduce inter-planner variability; dedicated efficiency analysis remains future work. The primary benefit demonstrated here is not necessarily a faster planning process, but the elimination of manual trial-and-error. By automating the iterative adjustment of MaxEUD constraints, the framework ensures a standardized baseline that is independent of individual planner experience and effort. PTV coverage remained statistically unchanged, confirming that dose reductions did not compromise target irradiation. A lower Paddick Conformity Index reflects intentional dose redistribution rather than reduced precision: the algorithm promotes tangential beam geometries that decrease the low-dose bath by shifting dose away from critical structures, even at the cost of increased prescribed isodose volume. 

Mechanistically, iterative MaxEUD adjustment drives this tangential distribution, reducing high-dose exposure to central organs and, for several metrics, approaching or surpassing dose sparing reported for 3DCRT. More broadly, the results indicate that conventional optimization endpoints may leave achievable dose reductions unrealized. The framework therefore represents a geometry-agnostic strategy in which tangential-like dose shaping emerges from objective-function–driven optimization rather than explicit beam restriction: non-tangential beam directions are not excluded but naturally down-weighted as inefficient solutions. The choice of beam arrangement in this study reflects the clinical standard for breast cancer treatment at our institution, where extended arcs are preferred to provide the optimizer with a wide range of degrees of freedom. We acknowledge that several studies, including those on DL- and KB-based models^81,82^, suggest that restricted partial arcs may be superior in limiting the low-dose bath to peripheral tissues. A limitation of the present study is that it did not explicitly investigate or compare the use of small partial arcs within the automated framework. However, as demonstrated in our analysis of the optimized dose contribution (Fig. 3), our framework independently prioritizes tangential entries, effectively minimizing the dose to the contralateral side despite the large available arc. The resulting geometry is thus a mathematically driven compromise that prioritizes the sparing of central OARs (heart and lung).

Robustness analyses demonstrate that the framework’s performance is highly stable across varying anatomies, with dose reductions remaining independent of PTV volume and inter-patient variability decreasing markedly. This consistent convergence toward objective-function saturation suggests that the process is primarily governed by optimization dynamics rather than contouring details. However, robustness does not imply unrestricted generalizability. The atypical funnel chest case shows that in challenging geometries, the automated process reaches a mathematical limit. At this point, professional intervention is essential to manage clinical trade-offs: the physicist must either (1) manually re-balance OAR sparing based on institutional constraints or (2) the framework must be expanded to include hard ’Clinical Goals’ for anatomical outliers. Consequently, the script provides a high-quality baseline while leaving final strategic prioritization to the expert.

The dosimetric improvements achieved by our OFV-guided framework align with and extend the findings of recent literature on deep learning (DL)-based automation in RayStation. For instance, van de Sande et al. demonstrated that DL dose-prediction models could achieve mean dose reductions for the heart and ipsilateral lung of approximately $0.5~\mathrm{Gy}$, even in complex, locally advanced breast cancer cases^45^. While our cohort consisted of standard breast cancer cases with generally lower baseline doses, the automated approach still achieved a substantial and highly significant reduction in mean heart dose (from 3.09 Gy to 2.34 Gy; $\Delta$ = 0.75 Gy). While absolute dose savings are not directly comparable across different patient cohorts, these results underscore the potential of automation to surpass manual planning. A key advantage of our iterative approach is its independence from historical training data. While DL models aim to emulate expert-level planning, their performance is inherently capped by the quality of the training ’gold standard’. In contrast, our OFV-guided framework systematically explores the individual optimization space until mathematical saturation is reached. This objective approach can identify sparing potential that might remain untapped by data-driven models, which may inadvertently reproduce sub-optimal patterns or institutional biases present in their training sets.

The magnitude of dose reductions in breast cancer treatment suggests implications for risk modelling^3,83^. Existing comparisons between VMAT and 3DCRT often assume higher organ doses than achieved here; updated modelling may alter projected long-term risk differentials. In particular, reductions in mean heart dose are clinically relevant given the absence of a threshold for radiation-induced cardiotoxicity and the linear relationship between mean dose and coronary events^10^. Reduced cardiac and pulmonary exposure may lower long-term toxicity risk based on epidemiological data and NTCP modelling^84–88^.

Use of conventional fractionation ensured comparability with established dosimetric literature. Because the optimization operates on physical dose convergence rather than fractionation-specific endpoints, relative OAR dose reductions are expected to translate proportionally to hypofractionated regimens, which warrants dedicated evaluation.

Finally, methodological robustness was emphasized over population generalization. While OFV are TPS-specific, reliance on relative OFV trends supports conceptual transferability. The core principle–optimization control based on OFV saturation–is system-independent, whereas the empirically derived MaxEUD coefficients represent institution-specific calibration. These parameters can be re-derived through limited sensitivity analysis, preserving general applicability of the underlying optimization concept.

## Conclusion

This study introduces an automated VMAT planning approach for breast cancer that leverages the OFV to systematically explore the physical limits of dose sparing. By implementing a Python-based workflow that iteratively adapts MaxEUD constraints until mathematical saturation is reached, we demonstrated that it is possible to significantly reduce the dose burden to healthy tissues (p<0.001) without compromising target volume coverage.

Our findings reveal that this OFV-guided optimization leads to a substantial reduction in mean doses to the heart, lungs, and contralateral breast compared to manual clinical planning. By emulating a more tangential dose distribution, the approach effectively minimizes high-dose volumes in critical organs. Furthermore, the significant reduction in monitor units and the decrease in the low-dose bath (V_2Gy_ and V_5Gy_) suggest a potential for minimizing the risk of secondary radiation-induced malignancies, although the specific impact of small partial arcs was not investigated. While we did not explicitly measure planning time, the primary advantage demonstrated here is the automation and standardization of the iterative optimization process.

In conclusion, this automated framework provides a robust and mathematically grounded method for breast cancer radiotherapy that can be translated to other clinical sites. While the algorithm demonstrated stability across the evaluated patient cohort, the results in cases of atypical anatomy highlight that such automated workflows are best utilized as baselines to be further refined by clinical experts. By reducing inter-planner variability and systematically exploring the optimization space, this approach offers the potential to establish a more reproducible standard for plan quality within the scope of VMAT breast radiotherapy. This standardized foundation could furthermore serve as a consistent basis for training future machine-learning models, mitigating the biases and variability typically associated with human-driven treatment planning.

## Data Availability

”The datasets generated and analysed during the current study are available from the corresponding author on reasonable request.”
